# Image-Based Sexual Abuse Associated Factors: A Systematic Review

**DOI:** 10.1007/s10896-023-00557-z

**Published:** 2023-04-25

**Authors:** Maria Noemi Paradiso, Luca Rollè, Tommaso Trombetta

**Affiliations:** Department of Psychology, University of Turin, Via Verdi 10, 10124 Turin, Italy

**Keywords:** Image-Based Sexual Abuse, Upskirting, Deepfaking, Revenge Porn, Sextortion, Technology, Systematic Review

## Abstract

**Purpose:**

Image-Based Sexual Abuse (IBSA) is a recently studied form of violence and abuse perpetrated using technology. This systematic review aims to examine and systematize studies exploring factors associated with IBSA (e.g., victimization, perpetration, and propensity to perpetrate).

**Method:**

Following the Preferred Reporting Items for Systematic Review and Meta-Analysis (PRISMA) statement, 17 articles were included.

**Results:**

The results of this study highlighted conceptual and methodological limitations in the literature on IBSA. Aside from these limitations, this systematic review identified factors associated with IBSA, focusing on four macro-areas: victimization, perpetration, propensity to perpetrate IBSA, and IBSA implications. The results demonstrated the role of psychological, relational, and social variables, although the effect sizes observed in the quantitative studies were small or in few cases moderate.

**Conclusions:**

These results suggest further research should be carried out to explore the multidimensionality of IBSA and its associated factors, which may assist in guiding interventions to promote preventive and rehabilitative methods to lower the prevalence of this crime and its consequences.

## Introduction

Technology has become an essential part of our social lives: a simple click is all it takes to break down geographical distances, to contact people on the other side of the world, and to expose and share one’s most intimate and private sphere. On the negative side, the rapid expansion of the World Wide Web has enabled digital technologies to be used as tools to facilitate sexual violence and has allowed “traditional” abusive behaviors (e.g. psychological, physical, and sexual violence) to take on the dramatic dimensions of the online world (Henry & Powell, 2018; Maddocks, 2018).

“Image-Based Sexual Abuse” (IBSA), defined by Powell and colleagues (2019) as the non-consensual creation, distribution, and/or threat to distribute intimate or sexual materials (i.e. images, videos, or texts), is one of the recently studied forms of violence and abuse perpetrated through technologies. Considering the broad spectrum of abusive behaviors that fall under this umbrella term, IBSA can be defined as a multidimensional construct that involves:

- the non-consensual taking of private sexual images, such as the capturing of intimate images under an individual’s clothing, also known as “upskirting”, and the non-consensual transposition through artificial intelligence of one image onto a secondary one giving the illusion that the person depicted is involved in sexually explicit behavior, also called “deepfake pornography” (McGlynn & Rackley, 2017; Fido et al., 2022);
- the non-consensual distribution of nude or sexual images, also known as revenge pornography (RP), non-consensual pornography (NCP), non-consensual intimate image (NCII), cyber-rape, or involuntary porn (Citron & Franks, 2014; Maddocks, 2018). In this case, the perpetrator may distribute consensually created intimate materials (e.g., taken in the context of a romantic relationship) or non-consensually created intimate materials (e.g., taking hidden recordings of victims while they engage in sexual acts or while they are nude, or recording of rape or other forms of sexual assaults). (Citron & Franks, 2014); and
- the threat to distribute nude or sexual materials if the victim does not comply with certain requests, so-called “sextortion” (O’Malley & Holt, 2020; Powell et al., 2019).

Despite the increased interest in IBSA, little is known about its prevalence. It is challenging to establish an accurate estimate of the level of IBSA victimization and perpetration due to conceptual and methodological limitations (i.e., the absence of a uniform definition of the phenomenon, the heterogeneity of the samples, and the differences in capturing the experiences of the victims). Consequently, controversial results emerge. A recent survey by Powell et al. (2022) aimed to examine the extent, nature, and correlates of adults’ self-reported perpetration of IBSA across the UK, Australia, and, New Zealand, and found that 17.5% of participants had perpetrated some form of IBSA, of which 15.8% had taken non-consensually nude or sexual images, 10.6% had shared nude or sexual images and 8.8% had threatened to disseminate nude or sexual images without the consent of the person depicted.

Most studies in this area have focused on the non-consensual dissemination of intimate materials (Englander & McCoy, 2017; Ruvalcaba & Eaton, 2020; Karasavva & Forth, 2021; Reed et al., 2021; Walker et al., 2021; Van Ouytsel et al., 2020b; Dardis & Richards, 2022). Overall, perpetration rates for the non-consensual distribution of intimate images appear to range from 5.12% (Ruvalcaba & Eaton, 2020) to 16.37% (Walker et al., 2021), whereas victimization rates range between 8% (Ruvalcaba & Eaton, 2020) and 28.5% (Karasavva & Forth, 2021). Empirical literature also reveals that gender has a mixed effect on IBSA perpetration and victimization. On one hand, some studies (Dardis & Richards, 2022, Gamez-Guadix et al., 2015; Karasavva & Forth, 2021; Reed et al., 2016; Ruvalcaba & Eaton, 2020) demonstrated higher levels of victimization among women; on the other hand, Powell et al. (2019) suggested that IBSA might involve female and male victims at similar rates. In contrast, Borrajo et al. (2015), Priebe and Svedin (2012), and Walker and Sleath (2017) found higher victimization rates for males than females. Finally, no association between gender and perpetration or victimization was found by Walker et al. (2021).

Despite the conflicting results on the prevalence of IBSA, drawing on Kelly's definition of a continuum of sexual violence which states that "[…] the continuum of sexual violence ranges from extensions of the myriad forms of sexism women encounter every day through to the all too frequent murder of women and girls by men" (Kelly, 1988, p.90), many scholars (Harper et al., 2021; Maddocks, 2018; McGlynn & Rackley, 2017) have begun to refer to IBSA as a continuum of sexual abuse from catcalling to rape. According to these scholars (Harper et al., 2021; Maddocks, 2018; McGlynn & Rackley, 2017), an effective legal and policy response can be developed by recognizing the fact that ISBA is part of the broader phenomenon of sexual violence.

While describing IBSA as sexual violence underlines the sexualized nature of the harassment, the gendered nature of the phenomenon, the absence of consent, and the damage caused to women’s sexual autonomy and dignity (Citron & Franks, 2014; McGlynn et al., 2017), the preliminary prevalence data available thus far make it difficult to draw firm conclusions in this regard. Despite these limitations, by considering IBSA as a continuum, it is possible to recognize the connections and similarities encompassed within the wide range of abuses based on the creation, distribution, and/or threat to disseminate sexual materials.

Although the limitations in terms of defining IBSA appear to be reflected in the poor use of validated and reliable measures of assessing it, preliminary studies (Bates, 2017; Campbell et al., 2020; Champion et al., 2022; Karasavva et al., 2022; Pina et al., 2017; Trujillo et al., 2020; van Oosten & Vandenbosch, 2020; Zvi, 2022) have explored factors associated with IBSA victimization, perpetration, and propensity to perpetrate, both in terms of predictors and consequences, also highlighting the negative influence of IBSA on well-being and mental health (e.g. emotional trauma, poor mental health, job loss, feelings of shame, public humiliation, and self-blame).

However, as yet, no systematic review has been conducted on the topic. Useful clinical information can be obtained by reviewing the data on factors associated with IBSA, which can then be used to guide prevention and intervention programs for both perpetrators and victims. In addition, the findings of recent research on the topic can provide important insights to guide future research in the field.

Accordingly, this study aims to review and systematize research focused on factors (i.e., predictors and consequences) associated with IBSA victimization, perpetration, and propensity to perpetrate.

## Materials and Methods

### Data Source and Search Strategy

Two independent reviewers (MNP and TT) conducted the systematic review following the Preferred Reporting Items for Systematic Review and Meta-Analyses (PRISMA) statement (Moher et al., 2009; Page et al., 2021). The systematic review was carried out through EBSCO (Databases: APA PsycArticles, APA PsycInfo, CINAHL Complete, Family Studies Abstracts, Gender Studies Database, Medline, Sociology Source Ultimate, Violence & Abuse Abstracts). No time constraints were imposed on the database search. All articles published between the creation of the databases and the month of May 2022 were screened. The research was conducted in two steps. The first keyword combination used was: violence OR abuse OR aggression OR harassment AND domestic OR “gender-based” OR partner OR spouse OR husband OR wife OR couple AND cyber OR technology OR internet OR computer OR image OR photo OR picture OR web. More specific terms were included in the second keyword combination, namely: “revenge porn*” OR “non-consensual porn*” OR “deepfake” OR “sextortion” OR “upskirting”.

### Inclusion and Exclusion Criteria

The inclusion criteria for this systematic review were: a) original research papers, b) published in the English language, c) focused on factors associated with victimization, the actual perpetration, and the propensity to perpetrate IBSA (i.e. non-consensual creation, distribution and/or threat to share intimate materials), e) explained the non-consensual nature of the phenomenon. All studies that did not meet the inclusion criteria were excluded. Moreover, the following exclusion criteria were applied: a) meta-analyses, b) literature reviews and c) legal articles.

### Study Selection and Data Extraction

**Fig. 1:**
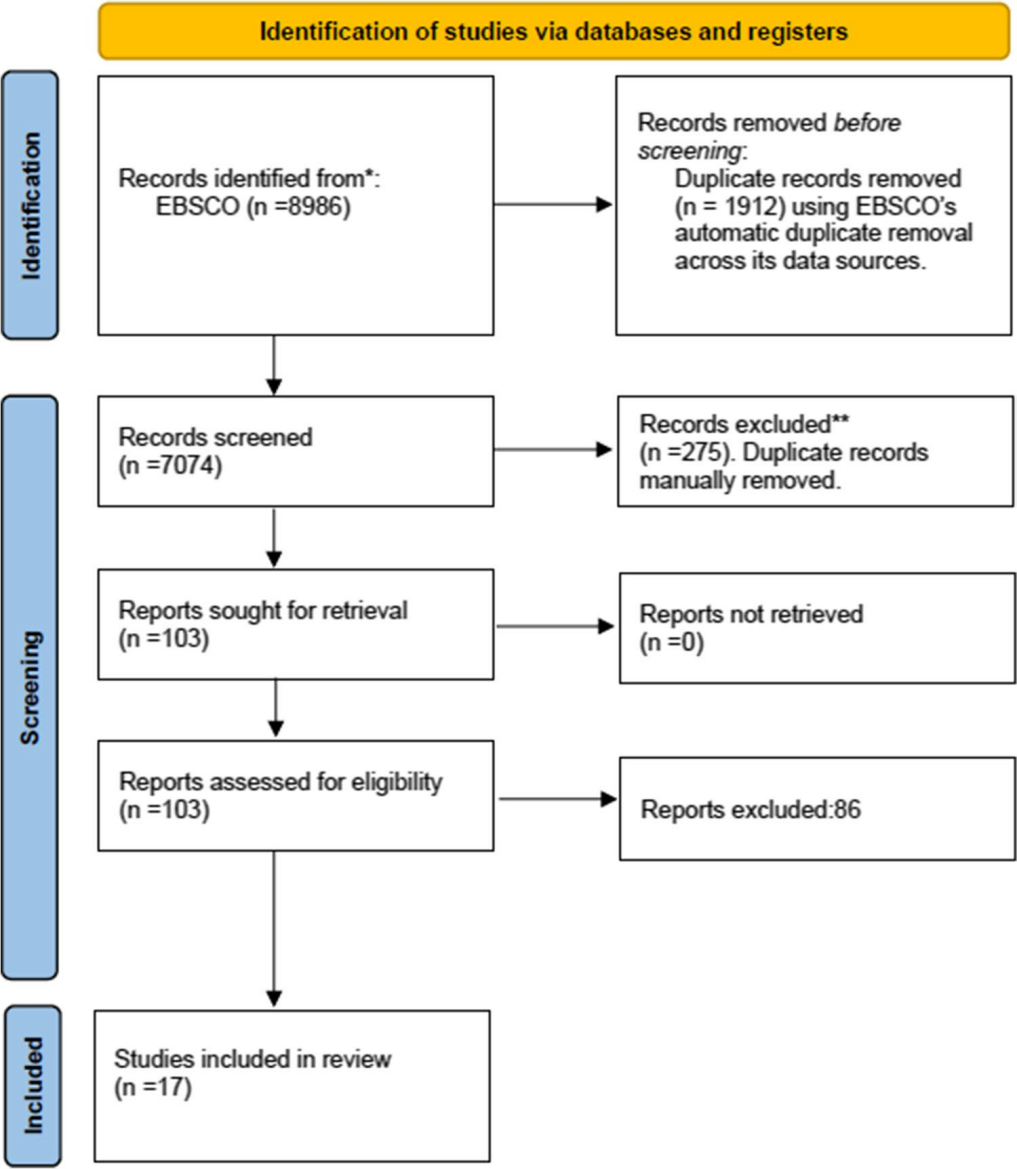
Flowchart of the selection procedure

The EBSCO search yielded 8986 articles and 7074 after removing duplicates. After screening the title and abstract, 103 articles were chosen for full-text review. Following full-text reading and application of the inclusion and exclusion criteria, 17 articles were included in this systematic review. Two independent reviewers (MNP and TT) examined the full text and extracted the data. Any disagreements were discussed between the reviewers in order to reach a unanimous decision. Figure 1 depicts a summary of the study selection process.

## Results

**Fig. 2:**
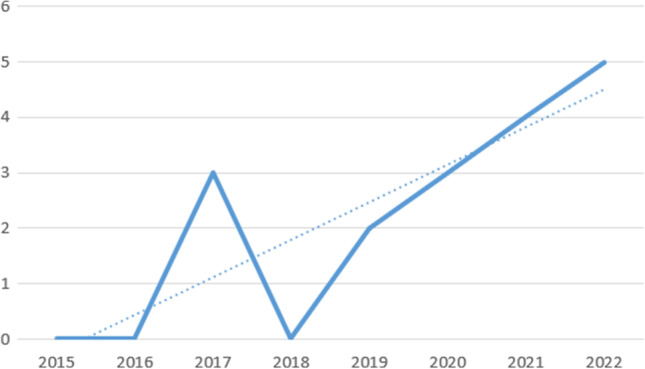
Graph of the number of publications across time. Note: The horizontal axis shows the period from which the publication spread, whereas the vertical axis shows the number of publications

Of the 17 studies included in this systematic review, most were conducted in the United States (5) or in Canada (3). Three studies were conducted in Australia, two in the United Kingdom, and two were multi-centric studies performed in the United Kingdom, Australia, and New Zealand. One study was carried out in the Netherlands, and one was conducted in Nigeria. From 2015 onwards, the interest shown by authors in IBSA grew. Notably, 3 studies were published from 2015 to 2018, while 14 articles were published from 2019 to 2022 (see Fig. 2 below). The public attention aroused by some IBSA cases has prompted scientific researchers to study the phenomenon only in recent years. In fact, although the first cases of IBSA date back to the 1980s, it is only since 2010—when Hunter Moore created a website for sharing intimate material without the consent of the people portrayed—that the phenomenon has gained more attention in the media and among lawmakers (Franks, 2017). The Moore case's high media profile turned the spotlight onto the IBSA phenomenon in all parts of the world. Since then, from Europe, with the European Women’s lobby’s “#Hernetherrights” campaign, to Latin America, with the campaign “La violencia en línea contra las mujeres en México” by the feminist collective Luchadoras, women’s rights organizations and feminist internet activists have promoted initiatives against IBSA and have called for the phenomenon to be criminalized (Maddocks, 2018).

The legislative landscape has also changed to incorporate action against IBSA. Starting from 2014 some European countries have been moving towards criminalizing IBSA. In 2014 Germany was the first European country to introduce a law against IBSA, followed by England and Wales in 2015, Scotland and France in 2016, and Italy in 2019 (European Institute of Gender Equality, 2017; Haynes, 2018; Greco & Greco, 2020). Several jurisdictions in the US have also followed the same path. While, prior to 2013, only three US states had enacted laws against IBSA, by the end of 2014 this had risen to 13, and by 2020 48 US states and Washington D.C. had legislated to combat the phenomenon (Franks, 2017; Wilkerson, 2022). This relatively new legislative and media evolution may explain the absence of studies on IBSA prior to 2015, which was when it first began to receive social and legal attention.

### Methodological Issues

Several methodological differences were found between the studies. Of the 17 articles included in this systematic review, eleven used a quantitative approach based on self-report instruments (Clancy et al., 2019; Dardis & Richards, 2022; Eaton et al., 2022; Karasavva & Forth, 2021; Karasavva et al., 2022; Pina et al., 2017; Powell et al., 2019, 2022; Ruvalcaba & Eaton, 2020; Short et al., 2017; van Oosten & Vandenbosch, 2020).

The dimensions of the investigated construct varied from study to study. In detail, 7 studies investigated the dimension of the non-consensual sharing of intimate material (also known as revenge porn, non-consensual dissemination or forwarding of sexts, non-consensual pornography, non-consensual dissemination of intimate materials or non-consensual distribution of sexually explicit images; Clancy et al., 2019; Karasavva & Forth, 2021; Karasavva et al., 2022; Pina et al., 2017; Ruvalcaba & Eaton, 2020; Short et al., 2017; van Oosten & Vandenbosch, 2020). One study focused on both sharing and threatening to share sexually explicit images or recordings without consent (Dardis & Richards, 2022), whereas another examined only the threat to distribute intimate material (i.e. sextortion; Eaton et al., 2022). Two articles considered the entire IBSA continuum (assessing each IBSA dimension independently; Powell et al., 2019, 2022),

From the studies assessing the non-consensual distribution of intimate images, one investigated perpetration (Clancy et al., 2019), two (Dardis & Richards, 2022; Short et al., 2017) investigated victimization, and another two (Karasavva & Forth, 2021; Ruvalcaba & Eaton, 2020) focused on both victimization and perpetration. The propensity to perpetrate non-consensual distribution of intimate images was investigated by three studies (Karasavva et al., 2022; Pina et al., 2017; van Oosten & Vandenbosch, 2020). Two research papers focused on victimization in the case of a threat to distribute intimate material (Dardis & Richards, 2022; Eaton et al., 2022), while the two studies that assessed the entire continuum of IBSA focused on perpetration (Powell et al., 2019, 2022).

To assess the occurrence and levels of IBSA victimization and perpetration, as well as the propensity to perpetrate IBSA, the quantitative articles we reviewed used a wide range of instruments. Table 1 (see below) summarizes the assessment tools used.

**Table 1:** Assessment tools

Nonconsensual distribution of intimate materials (perpetration)	Authors
Question developed by the authors ("*Have you ever knowingly shared a sexually explicit image or video of someone else without his/her consent?*")	Ruvalcaba and Eaton (2020)
*IBSA perpetration-distribute subscale* by Powell et al., (2019; "*Have you ever (since 16 years of age) distributed a nude or sexual image of another person without their consent?*")	Karasavva and Forth, (2021)
**IBSA continuum (perpetration)**	
Question developed by Powell et al., (2019; *Have you ever (since 16 years of age) taken, distributed, and/or threatened to distribute a nude or sexual image of another person without your consent?"*)	Powell et al. (2019)
Question developed by Powell et al., (2019; *Have you ever (since 16 years of age) taken, distributed, and/or threatened to distribute a nude or sexual image of another person without your consent?"*)	Powell et al. (2022)
**Nonconsensual distribution of intimate materials (victimization)**	
*Harassment and Revenge Porn Survey (HARP)*	Short et al. (2017)
Question developed by the authors (*"Has anyone ever shared a sexually explicit image or video of you without your consent?")*	Ruvalcaba and Eaton (2020)
Question developed by Eaton et al. (2017; "*Has anyone ever shared a sexually-explicit image or video of you without your consent?"*)	Dardis and Richards (2022)
*IBSA victimization-distribute subscale* by Powell et al., (2019; "*Have you ever (since 16 years of age) had a nude or sexual image of yourself distributed without your consent?")*	Karasavva and Forth, (2021)
**Threat to disseminate intimate materials (victimization)**	
Question developed by Eaton et al. (2017; "*Has anyone ever threatened to share a sexually-explicit image or video of you without your consent?"*	Dardis and Richards (2022)
Question developed by the authors (*"Have you ever been victim of sextortion?"*)	Eaton et al. (2022)
**IBSA continuum (victimization)**	
Question developed by Powell et al., (2019; *Have you ever (since 16 years of age) had a nude or sexual image of yourself taken, distributed and/or threatened to be distributed without your consent?*")	Powell et al. (2019)
Question developed by Powell et al., (2019; *Have you ever (since 16 years of age) had a nude or sexual image of yourself taken, distributed and/or threatened to be distributed without your consent?*")	Powell et al. (2022)
**Nonconsensual distribution of intimate materials (perpetration propensity)**	
Questions developed by the authors in accordance with the definition used by Dake et al. (2012; "*Has someone ever forwarded you a sexually explicit image *via* text or mobile app that was not originally intended for you?", "If yes, what were the reasons you decided to share the sext message with others*?")	Clancy et al. (2019)
*Sexual Image-based abuse myth acceptance* (SIAMA; Powell et al., 2019)	Karasavva and Forth (2021); Karasavva et al. (2022)
Revenge Porn Proclivity Scale (RPSS; Pina et al., 2017)	Karasavva et al. (2022); Pina et al. (2017)
Scenario and five items developed by the authors	van Oosten and Vandenbosch (2020)
**IBSA continuum (perpetration's propensity)**	
Sexual Image-based abuse myth acceptance scale by Powell et al. (2019)	Powell et al. (2019)
Sexual Image-based abuse myth acceptance scale by Powell et al. (2019)	Powell et al. (2022)

Five articles used a qualitative approach assessing IBSA victimization (Aborisade, 2021; Bates, 2017; Campbell et al., 2020; Henry et al., 2021; McGlynn et al., 2021). Of these articles, two investigated both the non-consensual dissemination of intimate images and the threat to share intimate materials (Bates, 2017; Campbell et al., 2020), one study investigated the first two dimensions of the IBSA continuum (i.e. non-consensual taking or non-consensual sharing of intimate material) within the macro-category of TFSV (Technology-Facilitated Sexual Violence) (Henry et al., 2021) and two focused on the entire continuum of IBSA (Aborisade, 2021; McGlynn et al., 2021). Among these, three articles used semi-structured interviews (Aborisade, 2021; Bates, 2017; Campbell et al., 2020) while two studies used unstructured interviews (Henry et al., 2021; McGlynn et al., 2021). Only one article followed a mixed-method approach (Champion et al., 2022). All articles were cross-sectional, except for one that adopted a longitudinal design (van Oosten & Vandenbosch, 2020).

With regard to the participants involved in the studies, most of the reviewed papers (14) focused on adults, mainly female, white, and well-educated (Aborisade, 2021; Bates, 2017; Campbell et al., 2020; Champion et al., 2022; Clancy et al., 2019; Dardis & Richards, 2022; Karasavva & Forth, 2021; Karasavva et al., 2022; McGlynn et al., 2021; Pina et al., 2017; Powell et al., 2019, 2022; Ruvalcaba & Eaton, 2020; Short et al., 2017). One article included immigrant and refugee women (Henry et al., 2021), and one focused on racial/ethnic minorities and LGBTQ individuals (Eaton et al., 2022). Finally, one study included both adolescents and young adults (van Oosten & Vandenbosch, 2020).

### Main Findings

The section below summarizes the results of the reviewed studies in four macro-areas: IBSA victimization and perpetration, propensity to perpetrate IBSA, and IBSA implications (see Table 2).

**Table 2:** Characteristics and conclusions of the included studies

Author(s)	Title	Country	Participants	Type(s) of IBSA assessed	Assessment tool(s)	Conclusions
Aborisade (2021)	Image-based sexual abuse in a culturally conservative Nigerian society: Female victims' narratives of psychosocial costs	Nigeria	N = 27 (F)	Image-based Sexual Abuse continuum	Semi-structured interviews	Fear, brunch of trust, and self-blame were experienced by victims who received threats to share intimate material from their intimate partners. Mental stress, paranoia, anger, guilt, depression, and suicidal ideation were experienced by victims of nonconsensual distribution of their intimate material. Among victims who used to be active on social media, many turned off their accounts or opened new ones with fake identity. All participants were blamed after the release of their intimate images and/or videos, expecially because they engaged in premarital sex, they shared intimate materials with their partners and because they were irresponsible while naked. Some of them dropped out or were expelled by their schools or lost their job
Bates (2017)	Revenge Porn and Mental Health: A qualitative analysis of the mental health effects of revenge porn on female survivors	Canada	N = 18 (F)	Nonconsensual distribution of intimate material and Threat to share intimate material	Semi-structured interviews	Under mental health, participants experienced breach of trust, PTSD, anxiety and depression, lower levels of self-esteem and confidence, loss of control over their body. Under negative coping mechanism, participants engaged in avoidance, denial, excessive alcohol consumption and, being obssessed over their own victimization. Under positive coping mechanism, participants sought help from a therapist, started to educate others about revenge porn, relied on the support of relatives and friends, tried to start a normal life again, seeked religion and, started to laugh about what happened
Campbell et al. (2020)	Social anxiety as a consequence of non-consensually disseminated sexually explicit media victimization	U.S	N = 17 (F = 15; M = 2)	Nonconsensual distribution of intimate material	Semi-structured interviews	As a consequence of the non-consensual dissemination of sexually explicit media participants reported fear of going out in public, fear of engaging in relationships, fear of looking for job, fear of seeking help and, feelings of depression and anxiety
Champion et al. (2022)	Examining the gendered impacts of technology-facilitated sexual violence: A mixed-methods approach	U.S	N = 333 (F = 210)	Image-based Sexual Abuse continuum	Modified version of the Technology-facilitated sexual violence victimization scale (TFSV-V; Powell & Henry, 2016); open-ended questions developed by the authors, Technology-facilitated sexual violence experience impact scale developed by the authors; semi-structured interviews	Participants experienced online image-based sexual abuse reported high levels of stress, anxiety, depression, negative interpersonal impact, negative consequences at school or work, moderate to extreme levels of suicidal ideation and problematic alcohol use and, suffered from byllying, rumors or gossip. They also reported loss of control over their images, privacy, and sexuality and fear of future re-victimization
Clancy et al. (2019)	The dark side of sexting- Factors predicting the dissemination of sexts	Australia	N = 505 (F = 338; M = 159; did not provide their gender = 8)	Nonconsensual distribution of intimate material	Questions developed by the authors in accordance with the definition used by Dake et al. (2012)	Age was not associated with perpetration. Machiavellianism, psychopathy and narcissism traits increased the likelihood of perpetration. Male participants agreed more than female participants that the nonconsensual distribution of intimate images would have increased their social status. Experiences of nonconsensual distribution victimization reduced the likelihood to perpetrate it. being sexually active and receiving a sext that was not originally destinated to the participant were positive predictors of perpetration
Dardis & Richards(2022)	Nonconsensual distribution of sexually explicit images within a context of coercive control: Frequency, characteristics, and associations wih other forms of victimization	U.S	N = 496 (F = 381; M = 108; non-binary = 5; self-defined = 1; prefer not to answer = 1)	Nonconsensual distribution of intimate material and Threat to share intimate material	Questions developed by Eaton et al. (2017)	In consensual distribution of sexually explicit images cases, women who had a romantic relationship with the perpetrator had a greater likelihood to experienced interpersonal violence such as psychological abuse, physical abuse, sexual abuse and in-person unwan ted pursuit behaviors, but not cyber unwanted pursuit behaviors, compared to women who were abused by a non-romantic partner. In sextortion cases, being in a romantic relationship decreased the likelihood to experience threats to distribute intimate material, compared to not being in a romantic relationship. However, receiving threats in a romantic relationship is associated with a greater likelihood to experience interpersonal violence
Eaton et al. (2022)	The relationship between sextortion during COVID-19 and pre-pandemic intimate partner violence: A large study of victimization among diverse U.S. men and women	U.S	N = 2006(F = 1051)	Threat to share intimate material	Question developed by the authors	In sextortion cases during the pandemic, age was a significant and negative predictor of victimization. Ethnic differences were significant only for women but not for men, specifically Native Alaskan individuals or Indigenous North American or African American individuals suffered more the threat to share intimate material compared to White participants during covid-19. LGBTQ participants had a greater risk of experiencing sextortion during pandemic compared to heterosexual participants. Having experienced physical or psychological IPV prior to covid-19 pandemic was not significant predictive of victimization during pandemic, whereas having suffered sexual IPV prior to the pandemic increased the likelihood of IBSA victimization. This relation did not differ between men and women
Henry et al. (2021)	Technology-facilitated domestic violence against immigrant and refugee women: A qualitative study	Australia	N = 29 (F)	Image-based Sexual Abuse continuum	Unstructured interview	Immigrants and refugee women reported lack of social support due to the due to family and religious beliefs about sex and privacy. Poor English communication, poor digital skills, and the inability to provide evidence of IBSA have been identified as specific barriers to help-seeking process. Having experienced IBSA has led to negative academic and work-related consequences
Karasavva & Forth (2021)	Personality, attitudinal, and demographic predictors of non-consensual dissemination of intimate images	Canada	N = 810 (F = 589)	Nonconsensual distribution of intimate material	IBSA victimization -distribute subscale and IBSA perpetration-distribute subscale by Powell et al. (2019); Sexual Image-based abuse myth acceptance (SIAMA; Powell et al., 2019)	Males had lower likelihood to be victim of nonconsensual distribution of intimate images compared to women. LGBQ + participants had a greater likelihood to be victims of nonconsensual distribution of intimate images compared to heterosexual participants. All dark personality traits, except for Machiavellianism, constituted significant predictors of perpetration and victimization. Specifically, high levels of sadism and narcissism increased the likelihood to perpetrate nonconsensual distribution of intimate images, whereas high levels of psychopathy and sadism increased the likelihood of victimization. Acceptance of IBSA-related myths was a significant predictor of perpetration. Aggrieved and sexual entitlement positively correlated with perpetration. Not having perpetrated nonconsensual distribution of intimate images reduced the likelihood of having been a victim, and not having experienced nonconsensual distribution of intimate images victimization reduced the likelihood to perpetrate it
Karasavva et al. (2022)	From myth to reality: Sexual image abuse myth acceptance, the Dark Tetrad, and non-consensual intimate image dissemination proclivity	Canada	N = 810 (F = 583)	Nonconsensual distribution of intimate material	Sexual Image-based abuse myth acceptance (SIAMA; Powell et al., 2019); Revenge Porn Proclivity Scale (RPSS; Pina et al., 2017)	High levels of narcissism, psychopathy, and sadism were positively associated with nonconsensual distribution of intimate material endorsing enjoyment. IBSA myths that minimize/ excuse the perpetrator were not only accepted the most by male and heterosexual participants but were also significant predictors of nonconsensual distribution of intimate material and IBSA
McGlynn et al. (2021)	"It's torture for the soul": The harms of image-based sexual abuse	UK, Australia, and New Zealand	N = 75 (F = 67, M = 6, T = 1, Other = 1)	Image-based Sexual Abuse continuum	Unstructured interview	Victims portrayed isolation from their family, friends, from the society and the online world. They also became hyper vigilant in online contexts starting to constantly monitor pornography sites and social media to make sure their images were no longer disseminated, turned off their accounts and limited their online interactions
Pina et al. (2017)	The malevolent side of revenge porn proclivity: Dark personality traits and sexist ideology	U.K	N = 100 (F = 82, M = 16)	Nonconsensual distribution of intimate material	Revenge Porn Proclivity Scale (RPSS; Pina et al., 2017)	Personality traits such as Machiavellianism, psychopathy and narcissism increased the likelihood of perpetration. Sadistic tendencies were not linked to nonconsensual distribution of intimate material proclivity
Powell et al. (2019)	Image-based sexual abuse: The extent, nature, and predictors of perpetration in a community sample of Australian residents	Australia	N = 4053 (F = 2298, M = 1755)	Image-based Sexual Abuse	Questions developed by the authors; Sexual Image-based abuse myth acceptance scale by Powell et al. (2019)	Gender was a significant predictor of the entire IBSA continuum perpetration. Specifically, male participants had a greater likelihood to perpetrate IBSA compared to female participants. LGB participants had a greater likelihood of perpetration compared to the heterosexual participants. Participants with disability/assistance needs had greater likelihood to perpetrate IBSA compared to people without disability/assistance needs. Being a victim increased the likelihood of perpetrating each of the three forms of abuse. Engaging in digital manifestation of intimacy such as sexual self-image behaviors increased the likelihood of perpetration
Powell et al. (2022)	Perpetration of image-based sexual abuse: Extent, nature and correlates in a multi-country sample	UK, Australia, and New Zealand	N = 6109 (F = 3183, M = 2926)	Image-based Sexual Abuse	Questions developed by Powell et al. (2019); Sexual Image-based abuse myth acceptance scale by Powell et al. (2019)	Gender was a significant predictor of the entire IBSA continuum perpetration. Specifically, male participants had a greater likelihood to perpetrate IBSA compared to female participants. Participants living in New Zealand had a greater likelihood to perpetrate the abuse compared to participants living in the UK or in Australia. Participants with disability/assistance needs had greater likelihood to perpetrate IBSA compared to people without disability/assistance needs. IBSA myths that minimize/ excuse the perpetrator were not only accepted the most by male and heterosexual participants but were also significant predictors of nonconsensual distribution of intimate material and IBSA. Being a victim increased the likelihood of perpetrating each of the three forms of abuse. Dating someone online increased the likelihood of perpetration. Engaging in digital manifestation of intimacy such as sexual self-image behaviors increased the likelihood of perpetration
Ruvalcaba & Eaton (2020)	Nonconsensual pornography among U.S. adults: A sexual scripts framework on victimization, perpetration, and health correlates for women and men	U.S	N = 3044(F = 1644)	Nonconsensual distribution of intimate material	Questions developed by the authors	Women and LGBQ + victims had a greater likelihood to be victims of nonconsensual distribution of intimate material. Most of the victims did not ask for help
Short et al. (2017)	Revenge Porn: Findings from the Harassment and Revenge Porn (HARP) Survey-Preliminary results	U.K	N = 64 (F = 59)	Nonconsensual distribution of intimate material	Harassment and Revenge Porn Survey (HARP) developed by the authors	As a consequence of nonconsensual distribution of intimate material nervousness, anxiety, fear that something terrible may happen again, intrusive and disturbing memories of the incident, avoidance and denial, extreme sleep disorders, and engaging in self-harms behaviors were reported. To regain a sense of control over the abuse, victims changed their social media accounts or their phone number. Victims also reported difficulties in starting new relationships. Victims received support from their family and friends but perceived a lack of support from authorities and services in seeking a solution to stop the abuse. Most victims never asked for help. After the nonconsensual release of intimate images victims decided to change their jobs or have been fired or downgraded; additionally, the loss of work caused also financial problems, which were compounded by large expenses for legal and therapy costs
van Oosten& Vandenbosch (2020)	Predicting the willingness to engage in non-consensual forwarding of sexts: The role of pornography and instrumental notions of sex	Netherlands	N = 1947	Nonconsensual distribution of intimate material	Scenario and five items developed by the authors	Instrumental attitudes toward sex marginally moderated the relationship between pornography use and the propensity to perpetrate only in the context of a stranger, but not in the context of a friend or a current or ex-partner. In the context of a stranger the relationship between pornography use and propensity to perpetrate nonconsensual distribution of intimate material was significant among youth with low instrumental attitudes toward sex and stronger among youth with higher instrumental attitudes toward sex. Pornography use predicted the proclivity to engage in nonconsensual distribution of intimate material among youth with high levels of instrumental attitudes toward sex in the context of a friend and an ex-partner, whereas among youth with low levels of instrumental attitudes toward sex pornography use predicted the propensity to perpetrate nonconsensual distribution of intimate material just in the context of a dating partner. In the context of a relationship partner no significant prediction regarding the propensity to engage in nonconsensual distribution of intimate material have been found

### Perpetration and Propensity to Perpetrate

Within the context of this systematic review, five of the reviewed studies assessed perpetration (Clancy et al., 2019; Karasavva & Forth, 2021; Powell et al., 2019, 2022; Ruvalcaba & Eaton, 2020), and three studies assessed propensity to perpetrate (Karasavva et al., 2022; Pina et al., 2017; van Oosten & Vandenbosch, 2020).

***Gender:*** Karasavva et al. (2022) found that gender was not significant or predictive of the proclivity towards non-consensual distribution of intimate material. When considering the entire continuum of IBSA, gender was found to be a significant predictor of IBSA continuum perpetration in two studies (Powell et al., 2019, 2022). More specifically, males were more likely than females to perpetrate each dimension of the IBSA continuum.

***Age:*** In the study by Clancy et al. (2019) on the non-consensual distribution of intimate material, age was not associated with perpetration.

***Sexual orientation:*** In two studies (Powell et al., 2019; Ruvalcaba & Eaton, 2020) homosexual and bisexual sexual orientation were found to increase the likelihood of perpetrating both the non-consensual distribution of intimate material and the entire IBSA continuum. In contrast, Karasavva et al. (2022) found that sexual orientation was not associated with any propensity towards the non-consensual distribution of intimate material.

***Nationality:*** In the multi-country study on the IBSA continuum conducted by Powell et al. (2022) participants living in New Zealand were more likely to perpetrate this type of abuse than participants living in the UK or in Australia.

***Disability:*** When considering the entire continuum of IBSA, two studies found that participants with disability/assistance needs were more likely to perpetrate IBSA than people without disability/assistance needs (Powell et al., 2019, 2022).

***Personality traits:*** In Karasavva et al. (2022) sadism was positively associated with the approval of non-consensual distribution of intimate material, while this finding was not supported by Pina et al. (2017) with regard to the proclivity towards non-consensual distribution of intimate material. In two studies, all three dark personality traits (i.e. Machiavellianism, which include traits associated with manipulation, lack of empathy and morality; psychopathy; and narcissism) showed a positive relationship with the proclivity towards non-consensual distribution of intimate material (Pina et al., 2017) or non-consensual sharing of intimate material (Clancy et al., 2019), while in Karasavva and Forth (2021) this latter form of IBSA was only associated with psychopathy and narcissism. When adding two subscales of the proclivity towards non-consensual distribution of intimate material (i.e. approval and enjoyment), Pina et al. (2017) found a positive relationship with Machiavellianism, ambivalent sexism, and the approval of non-consensual distribution of intimate material, and a positive association between narcissism and the enjoyment of non-consensual distribution of intimate material. In the study by Karasavva et al. (2022) the enjoyment of non-consensual distribution of intimate material was instead related to sadism, narcissism, and psychopathy. Psychopathy was, on the other hand, not associated with the enjoyment or approval of non-consensual distribution of intimate material in Pina et al. (2017).

***Attitudinal characteristics:*** With regard to the non-consensual distribution of intimate material, instrumental attitudes toward sex marginally moderated the relationship between pornography use and the propensity to perpetrate only in the context of a stranger, but not in the context of a friend or a current or ex-partner (van Oosten & Vandebosch, 2020). Additionally, in the context of a stranger, the relationship between pornography use and propensity to perpetrate non-consensual distribution of intimate material was significant among young people with low instrumental attitudes toward sex and stronger among young people with higher instrumental attitudes toward sex. Pornography use predicted the proclivity to engage in the non-consensual distribution of intimate material among young people with high levels of instrumental attitudes toward sex in the context of a friend or an ex-partner, whereas pornography use among young people with low levels of instrumental attitudes toward sex predicted the propensity to perpetrate non-consensual distribution of intimate material just in the context of a dating partner. In the context of an intimate relationship, no significant prediction regarding the propensity to engage in non-consensual distribution of intimate material was found (van Oosten & Vandenbosch, 2020).

The acceptance of IBSA myths that minimize/excuse the perpetrator (e.g. statements claiming that “women should be flattered if a partner or ex-partner shows nude pictures of her to some close friends” or “a man shouldn’t get upset if his partner sends nude pictures of him to others” or “if a person sends a nude or sexual image to someone else, then they are at least responsible if the image ends up online”) were strongly associated with the propensity to perpetrate non-consensual distribution of intimate material (Karasavva et al., 2022), with the perpetration of non-consensual distribution of intimate material (Karasavva & Forth, 2021), and with the perpetration of the entire IBSA continuum (Powell et al., 2022). More specifically, Karasavva et al. (2022) demonstrated that being male and having high levels of psychopathy, Machiavellianism, and sadism were positively linked with IBSA myths that minimize/excuse the perpetrator, whereas being male, heterosexual, and having high levels of Machiavellianism were positively linked with IBSA-related myths that blame the victim.

Finally, with regard to the perpetration of non-consensual distribution of intimate material, Clancy et al. (2019) highlighted that male participants were more in agreement than their female counterparts that this perpetration would increase their social status. More specifically, holding the view that the non-consensual distribution of intimate material was a frequent and enjoyable practice increased the likelihood of perpetrating the abuse.

***Sexual behaviors:*** With regard to the non-consensual distribution of intimate material, being sexually active and receiving a sext that was not originally intended for the participant were positive predictors of perpetration (Clancy et al., 2019). For each IBSA dimension, on the other hand, Powell et al. (2022) found that dating someone online increased the likelihood of perpetration. Additionally, engaging in the digital manifestation of intimacy, such as sending sexual self-images, increased the likelihood of perpetration in two studies (Powell et al., 2019, 2022).

### Victimization

This systematic review considered four studies that assessed victimization (Dardis & Richards, 2022; Eaton et al., 2022; Karasavva & Forth, 2021; Ruvalcaba & Eaton, 2020).

***Gender:*** With regard to non-consensual distribution of intimate material, gender was predictive of victimization in one study (Karasavva & Forth, 2021). More specifically, males were less likely to be the victim of non-consensual distribution of intimate images than women. In contrast, one study found that men were more likely to be the recipients of a threat to share intimate material (Eaton et al., 2022).

***Age*:** Considering the threat to distribute intimate material as an outcome, participants aged between 18–29 were more likely to experience this abuse during the pandemic compared with individuals aged 30–40, 41–64, and over 65. (Eaton et al., 2022).

***Sexual orientation:*** Sexual orientation was associated with IBSA victimization in three studies. In particular, LGBQ + participants were more likely to be victims of non-consensual distribution of intimate material (Karasavva & Forth, 2021; Ruvalcaba & Eaton, 2020) and to be the recipients of threats to share intimate material (Eaton et al., 2022).

***Ethnicity:*** Native Alaskans or Indigenous North Americans or African Americans were found to suffer more from the threat to distribute intimate material compared to White participants during the COVID-19 pandemic in the study by Eaton et al., 2022. However, this relationship was significant only for women and not for men.

***Personality traits:*** In Karasavva and Forth’s (2021) study on the non-consensual distribution of intimate material, high levels of psychopathy and sadism increased the likelihood of victimization.

***History of violence victimization:*** Having a history of IBSA victimization was associated with the perpetration of non-consensual distribution of intimate images in two studies (Clancy et al., 2019; Karasavva & Forth, 2021) as well as with the perpetration of the other three dimensions of the IBSA continuum in two other studies (Powell et al., 2019, 2022). With regard to the threat to share intimate materials, Eaton et al. (2022) found that the fact of having experienced physical or psychological intimate partner violence prior to the COVID-19 pandemic was not significantly predictive of victimization during the pandemic, whereas the fact of having suffered sexual IPV prior to the pandemic increased the likelihood of victimization. In addition, the strength of this connection did not differ between men and women. In the case of non-consensual distribution of intimate material, women who had a romantic relationship with the perpetrator were more likely to experience interpersonal violence, such as psychological abuse, physical abuse, sexual abuse, and in-person unwanted pursuit behaviors, but not cyber unwanted pursuit behaviors, compared to women who were abused by a non-romantic partner (Dardis & Richards, 2022). With regard to cases involving the threat to disseminate intimate material, persons in a romantic relationship were less likely to experience threats to distribute intimate material, compared to those not in a romantic relationship. However, receiving threats in a romantic relationship was associated with a greater likelihood of experiencing interpersonal violence (Dardis & Richards, 2022).

### IBSA Implications

***Mental health:*** Five studies identified negative consequences of IBSA (Aborisade, 2021; Bates, 2017; Campbell et al., 2020; Champion et al., 2022; Short et al., 2017), including anxiety, highlighted in one study regarding the non-consensual distribution of intimate material (Short et al., 2017), in two referring to the non-consensual sharing and threat of sharing intimate material (Bates, 2017; Campbell et al., 2020), and in one study assessing the entire IBSA continuum (Champion et al., 2022); depression, described in two studies assessing both the non-consensual distribution and the threat to share intimate material (Bates, 2017; Campbell et al., 2020), and in two studies assessing the entire IBSA continuum (Aborisade, 2021; Champion et al., 2022), and PTSD, highlighted in only one study on non-consensual distribution and the threat to share intimate material (Bates, 2017). Self-harm was described in one study assessing the non-consensual distribution of intimate material (Short et al., 2017); suicide attempt, in one study assessing the non-consensual distribution and the threat to distribute intimate material (Bates, 2017), and in one study assessing IBSA continuum (Aborisade, 2021); and suicidal ideation was found in two studies assessing the IBSA continuum (Aborisade, 2021; Champion et al., 2022). Stress was identified in one study assessing the non-consensual distribution of intimate material (Short et al., 2017), and in two studies assessing the IBSA continuum (Aborisade, 2021; Champion et al., 2022). Anger was found in one study assessing the non-consensual distribution of intimate material (Short et al., 2017), and in one study assessing the IBSA continuum (Aborisade, 2021). Sleep disorders were described in one study on the non-consensual distribution of intimate material (Short et al., 2017) and in one study on the non-consensual distribution and the threat to share intimate material (Bates, 2017). Finally, negative alcohol use was found in one study on the IBSA continuum (Champion et al., 2022).

***Self-esteem and loss of control:*** On one side, negative changes in self-confidence were highlighted in one study assessing both the non-consensual distribution and the threat to share intimate material (Bates, 2017), whereas negative changes in self-esteem were found in one study related to the entire IBSA continuum (Aborisade, 2021). On the other side, high levels of loss of control over one's own body, images, sexuality, and the online world were reported by victims of both non-consensual distribution and threat to disseminate intimate images (Bates, 2017) and by victims of IBSA (Champion et al., 2022). In addition, most victims of non-consensual sharing of intimate material changed their social media accounts (Short et al., 2017). In Short et al. (2017), victims of non-consensual sharing of intimate material changed their phone number (Short et al., 2017) and in two other studies, assessing the entire IBSA continuum, victims who were active on social media became hyper-vigilant in online contexts, constantly monitoring pornography sites and social media platforms to ensure that their images were no longer disseminated (Aborisade, 2021), and they also turned off their accounts and limited their online interactions (McGlynn et al., 2021).

***Engaging in relationships:*** Trust issues and difficulties in forming new romantic relationships and making new friends were highlighted in cases of non-consensual distribution of intimate material by Short et al. (2017), in cases of threats to share intimate material by Campbell et al. (2022) and in cases relating to the entire IBSA continuum by Aborisade (2021).

***Social support and isolation:*** Receiving social support from family and friends was highlighted in two studies, one focused on non-consensual distribution of intimate images (Short et al., 2017), and one concentrating on both non-consensual distribution and the threat to share intimate material (Bates, 2017). Social withdrawal was identified in five studies (Aborisade, 2021; Campbell et al., 2020; Henry et al., 2021; McGlynn et al., 2021; Short et al., 2017); more specifically, isolation from family and friends was reported in one study assessing non-consensual distribution of intimate material (Short et al., 2017), in one study assessing both non-consensual distribution and the threat to share intimate material (Campbell et al., 2020), and in three studies assessing the entire IBSA continuum (Aborisade, 2021; Henry et al., 2021; McGlynn et al., 2021). In addition, Aborisade (2021) emphasized the lack of social support from family and friends due to cultural beliefs about sex in Nigerian society, whereas Henry et al. (2021) highlighted isolation from family and friends due to religious beliefs about sex and privacy in a sample of immigrant and refugee victims of the IBSA continuum.

***School/work-related issues:*** Both non-consensual distribution of intimate images and sextortion were associated with the loss of a job (Aborisade, 2021; Bates, 2017; Campbell et al., 2020; Short et al., 2017) or a job change that resulted in a fear of seeking a new one (Bates, 2017; Campbell et al., 2020). Additionally, the loss of work also caused financial problems, which were compounded by the huge expense incurred for legal representation and for therapy (Short et al., 2017). In addition, participants who experienced both non-consensual distribution of intimate images and sextortion reported a lack of concentration at work after the incident (Bates, 2017; Campbell et al., 2020). With regard to the consequences of the IBSA continuum in educational settings, in Aborisade’s (2021) study some participants dropped out of their old school and applied to enroll at another one or were expelled when the school authorities became aware of the incident. When considering the entire IBSA continuum, negative consequences on academic and employment levels were also reported by Champion et al. (2022) and by Henry et al. (2021).

***Help-seeking:*** Among the victims of non-consensual distribution of intimate materials and sextortion, two studies found that many did not seek help after the incident (Campbell et al., 2020; Ruvalcaba & Eaton, 2020), whereas one study on non-consensual distribution of intimate material (Short et al., 2017) demonstrated that victims sought help from the police, helpline services, website, chat room or social media administrators or the perpetrator him/herself to stop the abuse. In Henry’s IBSA continuum study (2021) where the participants were immigrants or refugees, specific barriers to help-seeking were highlighted, such as poor English communication, lack of digital skills, and the inability to provide evidence of IBSA.

## Discussions

The aim of this paper was to review and systematize studies focused on factors associated with IBSA (i.e. victimization, perpetration, and propensity to perpetrate IBSA). Based on the inclusion and exclusion criteria, 17 articles were included.

As the research on IBSA is still in its early stages, several conceptual and methodological issues emerged. Firstly, although it has not been systematized, the lack of consensus on the definition of the phenomenon was highlighted. Few of the reviewed studies employed a multidimensional definition of the phenomenon (Aborisade, 2021; Champion et al., 2022; Henry et al., 2021; McGlynn et al., 2021; Powell et al., 2019, 2022) while most of them (Bates, 2017; Campbell et al., 2020; Clancy et al., 2019; Dardis & Richards, 2022; Eaton et al., 2022; Karasavva et al., 2022; Karasavva and Forth, 2021; Pina et al., 2017; Ruvalcaba & Eaton, 2020; Short et al., 2017; van Oosten & Vandenbosch, 2020) preferred to focus on one or two IBSA behaviors, providing only a partial picture of a more complex phenomenon. Secondly, the differences in the operational definition of the construct and the lack of validated and reliable instruments, as well as the paucity of data on factors associated with IBSA has contributed to the fragmentation of the scientific literature on IBSA. Furthermore, also due to conflicting findings on the prevalence of IBSA, the gendered nature of IBSA needs to be explored further, with greater theoretical reflection on the topic in order to guide future research and to interpret the related results. In addition, considering the methodological quality of the studies included, the quantitative studies comprehensively described the sample, the variables included, and the data collection procedure. However, limitations emerged in the description of the psychometric properties of the assessment tools used and the data analysis techniques. Only three studies (Karassava et al., 2022; Karasavva & Forth, 2021; Pina et al., 2017) appeared to demonstrate good methodological qualities in terms of these latter dimensions. For qualitative studies, on the other hand, most adequately describe the sampling procedures and data analysis techniques. Finally, both the quantitative and qualitative studies reviewed demonstrated limitations in terms of the possible generalization of the results.

Nevertheless, many variables were found to be associated with IBSA victimization, perpetration, and propensity to perpetrate IBSA, although effect sizes observed in the quantitative studies ranged from small to moderate. These findings seem to indicate that many variables (e.g., psychological, relational, and social) can have a role in IBSA, as emerge for intimate partner violence (Hardesty & Ogolsky, 2020), and Same-Sex Intimate Partner Violence (Trombetta & Rollè, 2022). Accordingly, most of the considered socio-demographic variables were associated with IBSA perpetration and victimization, but not with the propensity to perpetrate IBSA. The factors of gender, age, sexual orientation (e.g., homosexual, and bisexual sexual orientation), nationality, and disability were related to IBSA perpetration, whereas the factors of gender, age, sexual orientation, and ethnicity were related to IBSA victimization. In particular, the finding that men are more likely to perpetrate IBSA than women (Karasavva et al., 2022; Powell et al., 2019, 2022) is in line with the current debate on the gendered nature of the phenomenon. The feminist perspective has suggested that IBSA is influenced by patriarchal discourse and practices (DeKeseredy & Schwartz, 2016); more specifically, IBSA is seen as a manner of “doing masculinity” in a cultural way (Messerschmidt, 1993). Moreover, this systematic review found that the acceptance of myths that minimize/excuse the perpetrator correlated positively with the propensity to perpetrate non-consensual distribution of intimate material (Karasavva et al., 2022), the perpetration of non-consensual distribution of intimate material (Karasavva & Forth, 2021), and the perpetration of the entire IBSA continuum (Powell et al., 2022). These results, although still preliminary, are in line with a previous systematic review on face-to-face sexual violence (Yapp & Quayle, 2018) which demonstrated that the acceptance of rape myths constituted a risk factor for perpetration.

With regard to victimization, conflicting results emerged. Karasavva and Forth (2021) found that victimization was higher among women, in cases of non-consensual distribution of intimate material, whereas in terms of the threat to share intimate material, Eaton et al. (2022) found that victimization was higher among men. Previous research on sexting (i.e. consensual sharing of sexually explicit material) showed that women were more likely to suffer both coercive and pressure sexting, suggesting that women were more vulnerable to this kind of behavior (Drouin et al., 2015; Van Ouytsel et al., 2020a, b); however, as highlighted by Patchin and Hinduja (2020), in a national survey of US young people, males were more likely to be both perpetrators and victims, so it is possible that those who have been offended are more likely to offend.

Sexual orientation was associated with both IBSA perpetration (Karasavva et al., 2022; Powell et al., 2019; Ruvalcaba & Eaton, 2020) and victimization (Eaton et al., 2022; Karasavva & Forth, 2021; Ruvalcaba & Eaton, 2020). More specifically, LGB individuals appear to be more likely to be perpetrators (Karasavva et al., 2022; Powell et al., 2019; Ruvalcaba & Eaton, 2020) or victims (Eaton et al., 2020; Karasavva & Forth, 2021; Ruvalcaba & Eaton, 2020) of IBSA than heterosexual people. One potential explanation for this result is that LGB people are more likely to engage in sexting behaviors (Van Ouytsel et al., 2020a, 2020b) and to use dating apps online (Johnson et al., 2017), which emerged as risk factors of IBSA. This does not mean that people who exchange intimate material online consensually or who engage in online dating perpetrate IBSA or are more frequent victims of IBSA; however, it may mean they are more vulnerable to the phenomenon (Karasavva & Forth, 2021).

Dark personality traits were positively correlated with IBSA perpetration, and propensity to perpetrate. Previous research (Moore & Anderson, 2019) confirmed a relationship between dark personality traits and the involvement in antisocial behaviors online. Therefore, as personality traits might increase an individual’s likelihood of committing IBSA, it may be important to examine them in clinical settings, although future studies are needed to confirm these preliminary findings.

Specific attitudinal characteristics (e.g., instrumental characteristics towards sex and pornography use) demonstrated a positive correlation with a propensity to perpetrate IBSA (van Oosten & Vandensbosch, 2020). Through social learning, those who watch pornography integrate the actions they see into their own sexual scripts (Braithwaite et al., 2015); furthermore, because pornography promoted instrumental attitudes towards sex, it appeared to increase the willingness to engage in IBSA (van Oosten & Vandensbosch, 2020).

Although IBSA does not only occur within intimate relationships (Walker & Sleath., 2017), in some cases it seemed to correlate with other forms of intimate partner violence. With regard to the threat to share intimate material, Eaton et al. (2022) found that the fact of having experienced sexual violence prior to the COVID-19 pandemic increased the likelihood of victimization, whereas in the case of non-consensual distribution of intimate material, women who had a romantic relationship with the perpetrator were more likely to experience interpersonal violence (Dardis & Richards, 2022). Although, to date, no study has assessed quantitatively the association between IBSA and intimate partner violence, Eaton et al. (2021) highlighted a link between them using the Power and Control Wheel theory by Pence and Paymar (1993), demonstrating the use of all eight metatactics also in the online world. More specifically, emotional abuse was the most frequently used metatactic in IBSA cases, while the tactic of isolation was the least frequent (Eaton et al., 2021). As noted by this systematic review, isolation may be a consequence of IBSA rather than a specific perpetration method. However, these results infer that IBSA can be included within the intimate partner violence continuum and highlight the role of technology in its perpetration (Walker & Sleath, 2017).

The most consistent results that emerged appear to relate to the implications of IBSA. According to the studies we reviewed, IBSA victims suffered from a wide range of negative psychological, relational, and social effects. The negative impacts encountered included anxiety (Bates, 2017; Campbell et al., 2020; Champion et al., 2022; Short et al., 2017), depression (Aborisade, 2021; Bates, 2017; Campbell et al., 2020; Champion et al., 2022), and PTSD (Bates, 2017). In terms of mental health symptoms, IBSA victims experienced consequences similar to those of sexual assault victims (Campbell et al., 2009; Yuan et al., 2006). In fact, according to the literature on sexual violence, sexual assault victims also developed fear and anxiety, major depressive disorder, substance abuse, and suicidality (Rothman et al., 2021). In addition, high levels of loss of control over their body, images, sexuality, and the online world have been reported by victims of both non-consensual distribution and threat to disseminate intimate images (Bates, 2017) and by victims of the entire IBSA continuum (Bates, 2017; Champion et al., 2022). The lack of control over their bodies and intimate materials resulted in hyper-vigilant behavior in digital contexts, constant monitoring of pornography sites, and online profile deletion (Aborisade, 2021; McGlynn et al., 2021). Similarly, victims of face-to-face sexual violence also experience a lack of control over their bodies, a sense of powerlessness, and a loss of identity as a result of the rape experience (Zihindula & Maharaj, 2015). In this systematic review, social withdrawal was evidenced in five studies (Aborisade, 2021; Campbell et al., 2020; Henry et al., 2021; McGlynn et al., 2021; Short et al., 2017). Aborisade (2021) also emphasized the lack of social support from family and friends due to cultural beliefs about sex in Nigerian society, whereas Henry et al. (2021) highlighted isolation from family and friends due to religious beliefs about sex and privacy in a sample of immigrant and refugee victims of the IBSA continuum. In addition, both non-consensual distribution of intimate images and sextortion were associated with the loss of a job (Aborisade, 2021; Bates, 2017; Campbell et al., 2020; Short et al., 2017) or a job change that resulted in a fear of seeking a new one (Bates, 2017; Campbell et al., 2020) after the disclosure of the abuse.

Finally, several aspects influenced the help-seeking process among victims of IBSA. Two studies (Campbell et al., 2020; Ruvalcaba & Eaton, 2020) found that in cases of non-consensual distribution and threat to share intimate material, victims did not seek any help. The main reasons why victims did not ask for help were that they felt judged (Campbell et al., 2020), and that they felt embarrassed (Ruvalcaba & Eaton, 2020) by what had happened. In contrast, one study (Short et al., 2017) highlighted that victims sought help from police, helpline services, website, chat room or social media administrators, or the perpetrator him/herself to stop the abuse. According to several authors (Aborisade, 2021; Henry et al., 2021; Karasavva et al., 2022) acceptance of IBSA myths, cultural norms, social isolation, and victim blaming limited the ability to seek help. For this reason, services and interventions aimed at raising awareness on technology-facilitated violence, at changing attitudes to reduce victim-blame, and at promoting bystander intervention need to be developed.

### Limitations

The main findings of this systematic review must be considered in view of the study’s limitations. Firstly, as this is not a meta-analysis, no statistical conclusions can be drawn from its findings. Secondly, given the small number of articles included, the results should be interpreted with caution. Thirdly, although the idea of this systematic review was to evaluate the factors associated with the three dimensions of IBSA, no article on the non-consensual taking of private sexual material was found or included. Finally, the search carried out in this review was restricted to studies published in English, excluding articles in other languages that might have provided a more comprehensive understanding of the phenomenon.

### Future Directions

In order to understand the complexity of the phenomenon and to reach an agreement on the possible dimensions that constitute IBSA, and on how to operationalize and measure them, it is crucial firstly to reach a consensus on the definition of the construct. This systematic review used the multidimensional definition proposed by Powell et al. (2019). As encouraging as this conceptualization appears to be, further research is required to assess its multidimensionality and to test whether risk factors or consequences vary across dimensions, also developing validated assessment tools, which are currently absent. More generally, rigorous studies using validated and reliable instruments, larger and more representative samples, and mixed design methods are required in order to deepen our understanding of the phenomenon.

Preventive interventions often suggest that risk management strategies should be employed in order to avoid falling into the IBSA trap. Nevertheless, like risk management strategies in cases of sexual abuse, strategies aimed at avoiding IBSA shift the responsibility for such incidents onto the victim, thus absolving the perpetrator (Bates, 2017). Instead, educational material that focuses on dissolving the myths surrounding IBSA may be more effective in reducing the propensity to perpetrate it (Karasavva et al., 2022). As shown by Martini and De Piccoli (2020), attempting to change attitudes in order to reduce victim blaming and promote bystander intervention could provide better support to victims.

## Conclusions

Digital tools promote infinite ways of holding relationships that, for the first time in history, are completely unrelated to the physical dimension. Moreover, in encouraging a certain degree of disinhibition, the Internet has led to experiences of new forms of intimacy. Beyond their many positive uses and applications, digital technologies also have negative aspects. The rapid growth of IBSA is a clear example of the dark side of the Internet. By reviewing and systematizing factors associated with IBSA victimization, perpetration, and propensity to perpetrate, this systematic review has highlighted several gaps in the existing scientific literature.

Firstly, methodological issues emerged from the articles reviewed. The lack of validated assessment tools, differences in the operational definition of the construct, and the paucity of data on factors associated with IBSA as well as the lack of possible generalization of the results emerged from the studies we reviewed, and the literature was fragmented. For these reasons, the results should be interpreted with caution.

Beyond the conceptual and methodological limitations, this systematic review highlighted several psychological, relational, and social factors associated to IBSA victimization, perpetration, and propensity to perpetrate although the effect size observed in the quantitative studies ranged between small and moderate. Accordingly, additional research is needed to explore the variables associated with IBSA. Interventions aimed at promoting preventive and rehabilitative methods to lower the prevalence of this crime and its consequences should also be developed.
